# Supplementary material for: Substorm expansion embedded in a global cycle of field-aligned currents and auroral electrojets
Source: Nat Commun. 2026 Feb 20;17:2970. doi: 10.1038/s41467-026-69753-x (PMC13035851; doi:10.1038/s41467-026-69753-x)
Supplement: Supplementary file 1 — Supplementary Information [file 41467_2026_69753_MOESM1_ESM.pdf]

Supplementary Information for "Substorm Expansion Embedded in a Global Cycle of Field-Aligned Currents and Auroral Electrojets"

Tonghui Wang[1,2], Lei Dai[1*], C. Philippe Escoubet[3], Walter Gonzalez[4], Yong Ren[1], Minghui Zhu[1], Shan Wang[5], Chi Wang[1], Xu Wang[1,2], Kailai Wang[1,2], Jinjuan Liu[6]

[1]State Key Laboratory of Space Weather, National Space Science Center, Chinese Academy of Sciences, Beijing, China.
[2]University of Chinese Academy of Sciences, Chinese Academy of Sciences, Beijing, China.
[3]European Space Research and Technology Centre, European Space Agency (ESA), Noordwijk, Netherlands.
[4]National Institute for Space Research (INPE), São José dos Campos, São Paulo, Brazil.
[5]Institute of Space Physics and Applied Technology, Peking University, Beijing, China.
[6]CMA–USTC Laboratory of Fengyun Remote Sensing, ,University of Science and Technology of China, Hefei, China.

*Corresponding author(s). E-mail(s): ldai@spaceweather.ac.cn;

Supplementary Fig. 1 presents the solar wind conditions and geomagnetic indices associated with a sequence of intense substorms embedded within the main phase of the 17 March 2015 geomagnetic storm. Following 12:00UT, IMF B_z remains persistently southward, fluctuating around −20nT to −30nT with brief excursions northward. This

prolonged period of strong southward IMF and fast solar wind drives a series of intense substorms. The first substorm reaches a peak intensity with SML dropping below −2000nT around 13:30UT. SML index in the expansion phase of subsequent substorms exhibit minimum values ranging from −1300nT to −2400nT.

Supplementary Fig. 2 and Supplementary Fig. 3 show the coordinated evolution of Region 1 FACs, ionospheric convection and local SML across MLT and MLAT during the second and third substorm.

Supplementary Fig. 4 and Supplementary Fig. 5 show the coordinated evolution of Region 1 FACs, and local SML across MLT and MLAT during the fifth cycle (6th substorm).

Supplementary Fig. 6, Supplementary Fig. 7, Supplementary Fig. 8 show that the cycle of current peaks identified in the main text appear to be common for many (20-30) substorms during non-storm time. In the second panel of these figures, the longitudinal motion of the SML peak in many events appears as discrete jumps, likely associated with the addition or decay of nightside DP-1 currents, consistent with Gjerloev et al. (2004).

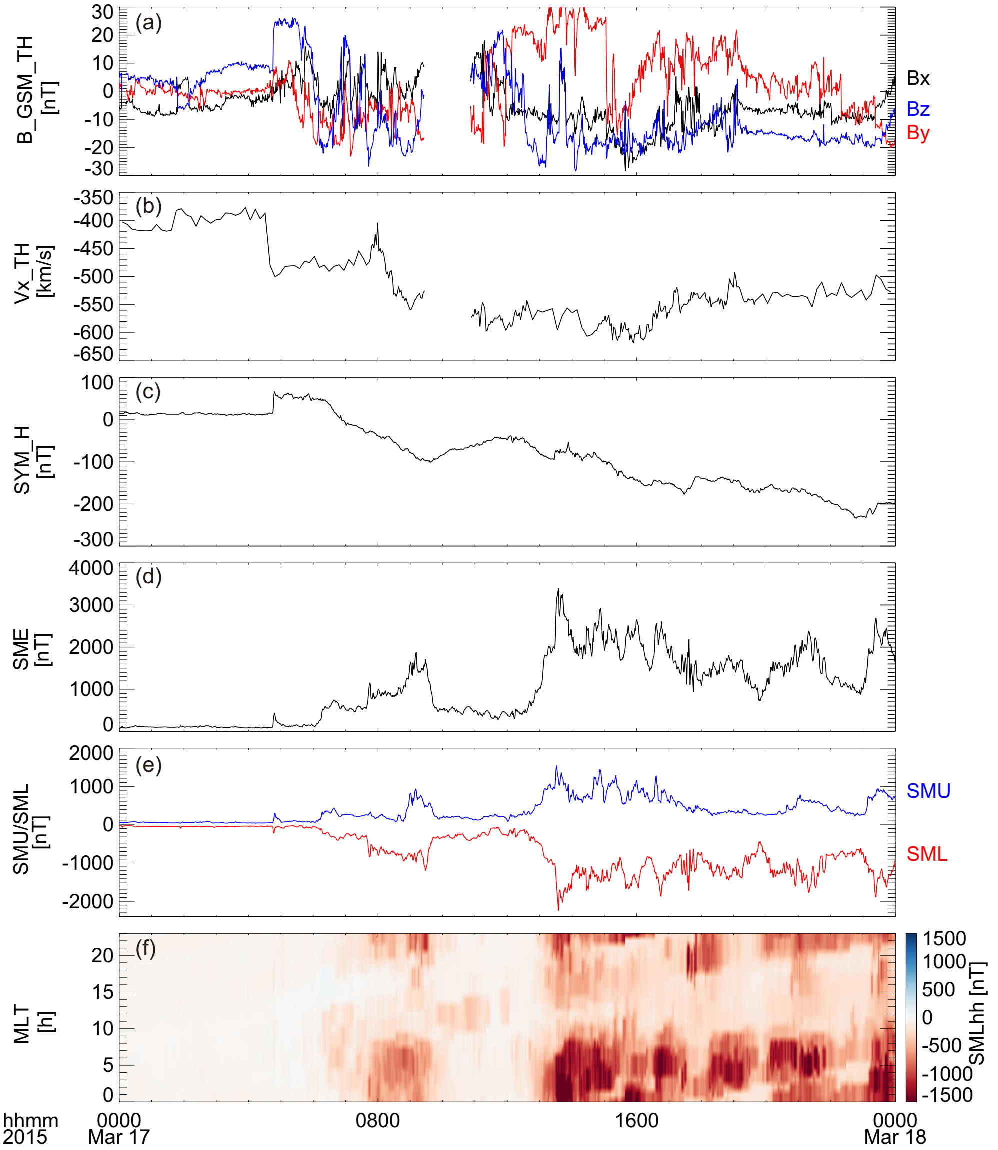

Supplementary Fig. 1 Overview of a series of intense substorms during the 17 March 2015 Storm. **a** The interplanetary magnetic field (IMF) in geocentric solar magnetosphere (GSM) coordinate system from THEMIS-B satellite. **b** Solar wind velocity V_x component in GSM from THEMIS. **c** SYM-H index; **d** SME index (SuperMAG version of AE index). **e** SMU (blue) and SML(red) index. **f** The distribution of regional SML index as a function of MLT and time. Source data are provided as a Source Data file.

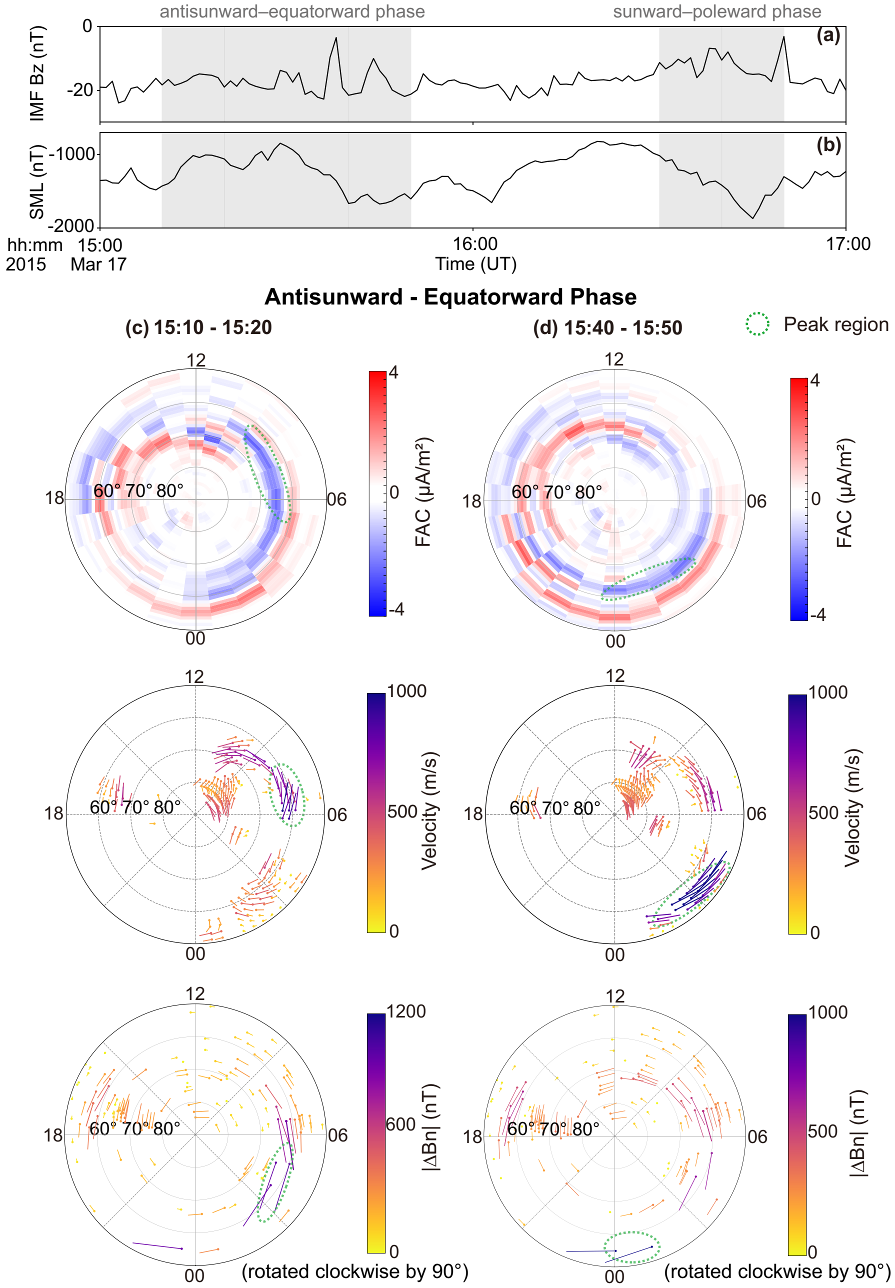

Supplementary Fig. 2 Coordinated evolution of Region 1 FACs, ionospheric convection, and local SML (westward AEJ) during 15:00 UT–17:00 UT on 17 March 2015. **a** IMF B_z and **b** SML index. The gray shadow represents the time covered by the event, divided into the antisunward-equatorward phase and the sunward-poleward phase. Snapshots of AMPERE-derived FACs (10-minute resolution), SuperDARN convection patterns (10-minute average), and local SML (10-minute median values of north–south B_N perturbations rotated by 90 degrees, characterizing the auroral electrojet) during the antisunward-equatorward phase at selected intervals: **c** 15:10–15:20 UT, **d** 15:40–15:50 UT. Red and blue indicate upward and downward FACs, respectively. Dawnside Region 1 FACs are downward (blue). The green dashed lines highlight the peak regions of the data. Source data are provided as a Source Data file.

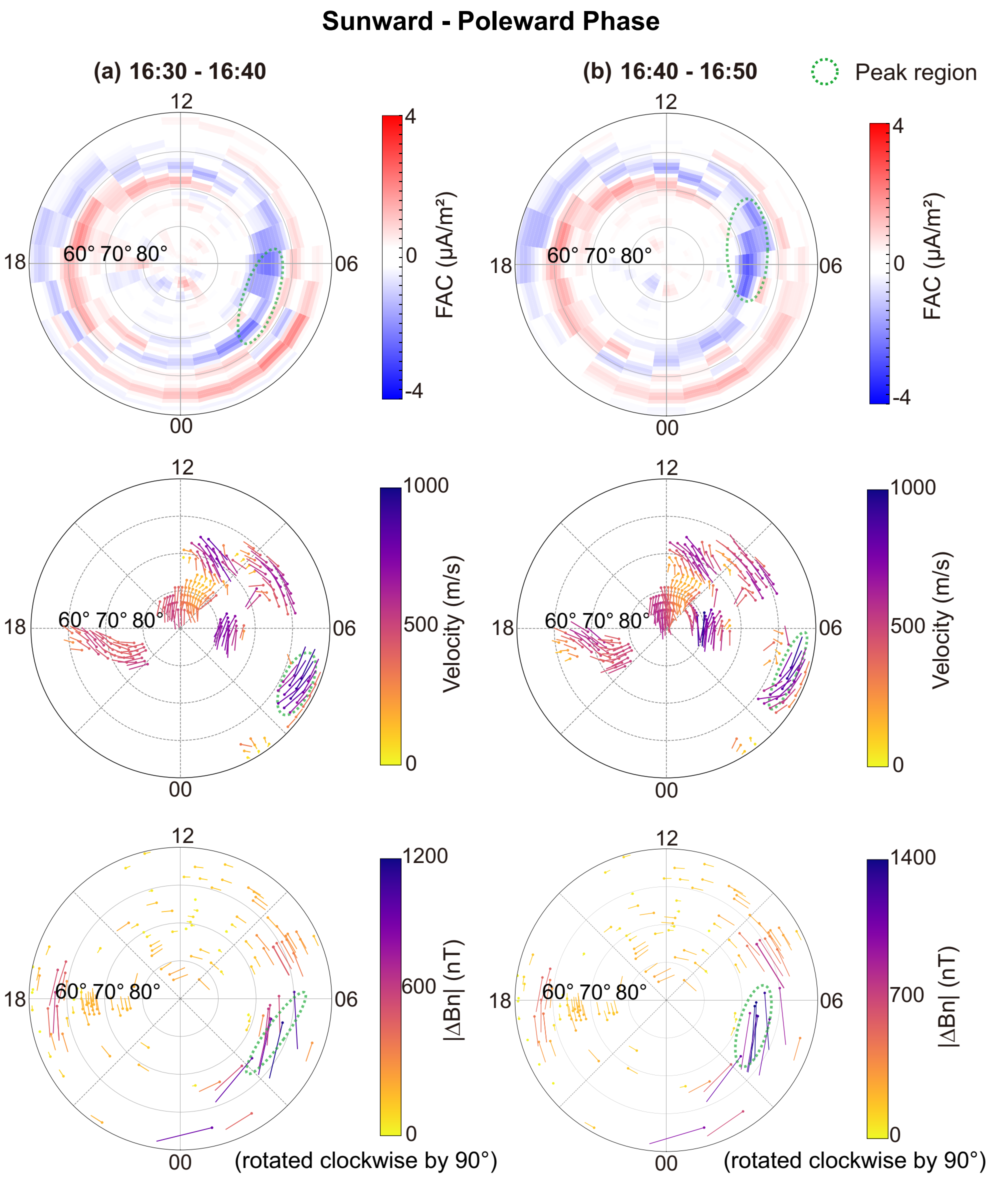

Supplementary Fig. 3 As a continuation of Supplementary Fig. 2 , evolution during the sunward-poleward phase. Snapshots of AMPERE-derived FACs (10-minute resolution), SuperDARN convection patterns (10-minute average), and local SML (10-minute median values of north–south B_N perturbations rotated by 90 degrees, characterizing the auroral electrojet) at selected intervals: **a** 16:30–16:40 UT, **b** 16:40–16:50 UT. Red and blue indicate upward and downward FACs, respectively. Dawnside Region 1 FACs are downward (blue). The green dashed lines highlight the peak regions of the data. Source data are provided as a Source Data file.

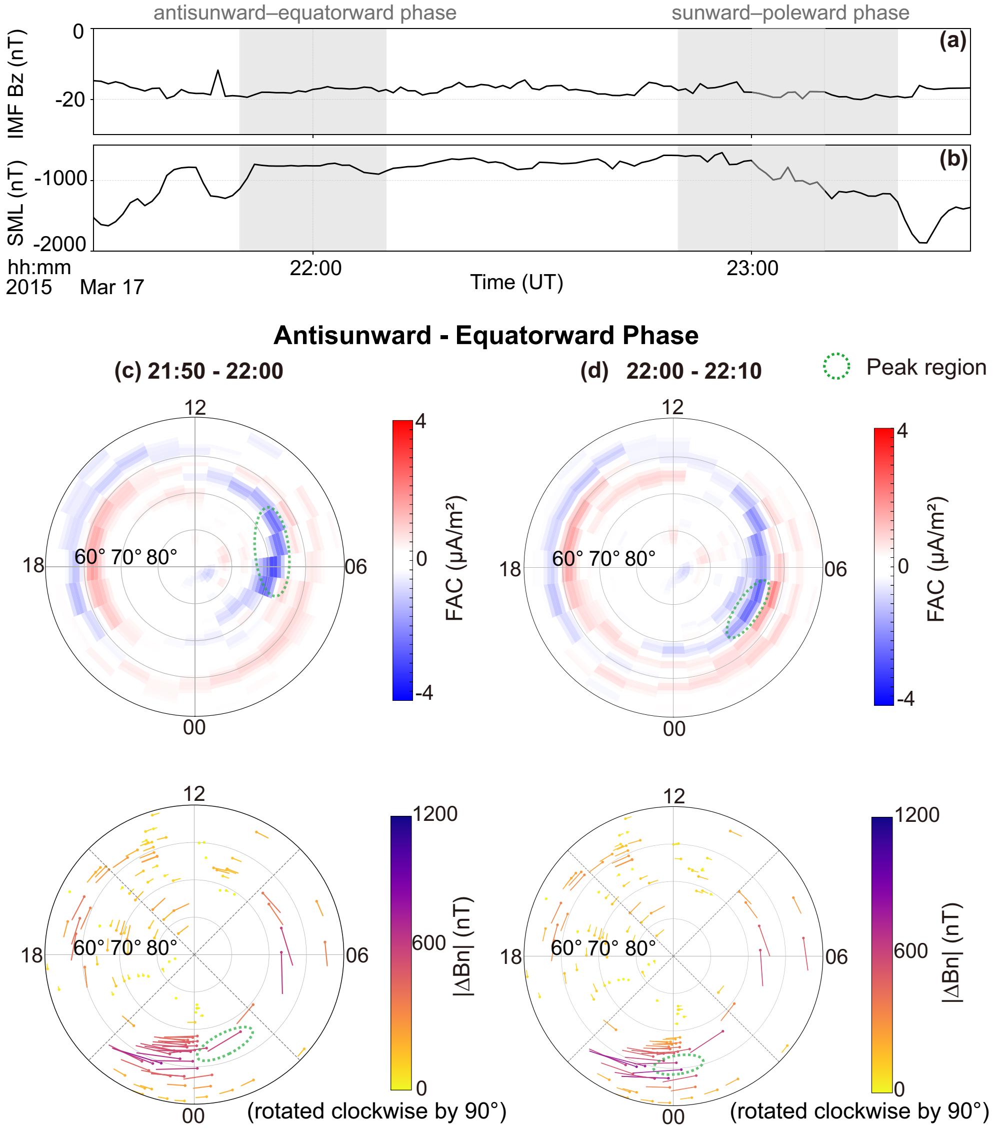

Supplementary Fig. 4 Coordinated evolution of Region 1 FACs, and local SML (westward AEJ) during 21:30 UT–23:30 UT on 17 March 2015. **a** IMF B_z and **b** SML index. The gray shadow represents the time covered by the event, divided into the antisunward-equatorward phase and the sunward-poleward phase. Snapshots of AMPERE-derived FACs (10-minute resolution), and local SML (10-minute median values of north–south B_N perturbations rotated by 90 degrees, characterizing the auroral electrojet) during the antisunward-equatorward phase at selected intervals: **c** 21:50–22:00 UT, **d** 22:00–22:10 UT. Red and blue indicate upward and downward FACs, respectively. Dawnside Region 1 FACs are downward (blue). The green dashed lines highlight the peak regions of the data. SuperDARN lack adequate coverage in the dawn sector for this interval. Source data are provided as a Source Data file.

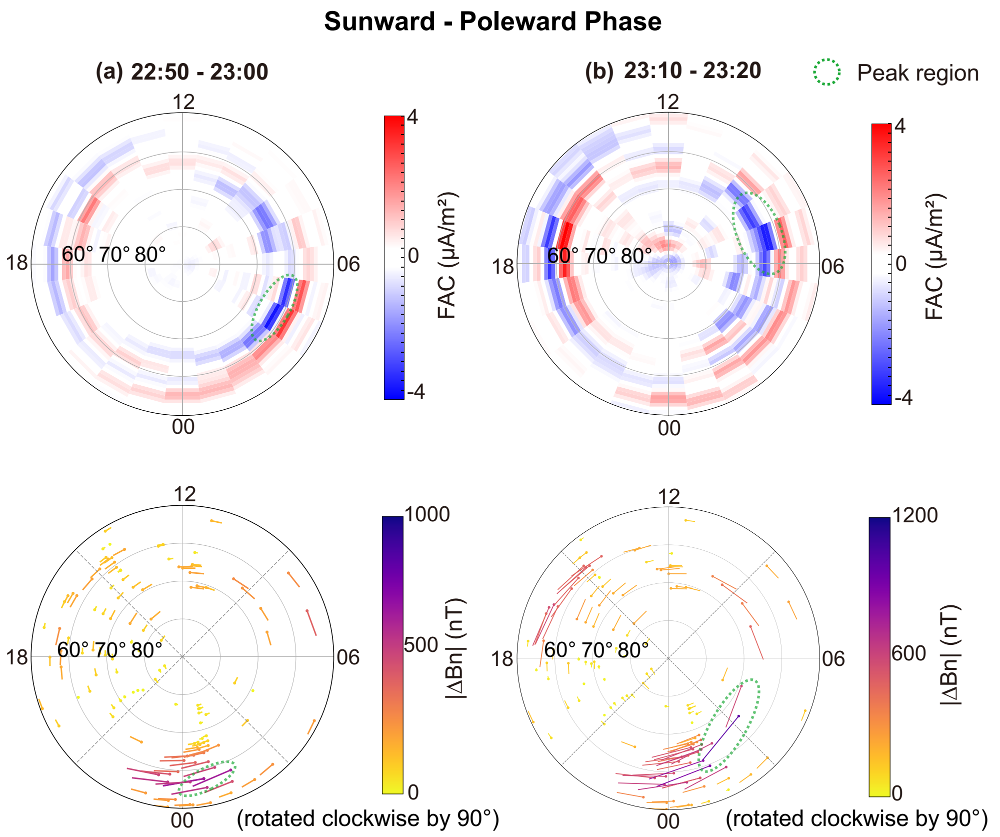

Supplementary Fig. 5 As a continuation of Supplementary Fig. 4 , evolution during the sunward-poleward phase. Snapshots of AMPERE-derived FACs (10-minute resolution) and local SML (10-minute median values of north–south B_N perturbations rotated by 90 degrees, characterizing the auroral electrojet) at selected intervals: **a** 22:50–23:00 UT, **b** 23:10–23:20 UT. Red and blue indicate upward and downward FACs, respectively. Dawnside Region 1 FACs are downward (blue). The green dashed lines highlight the peak regions of the data. SuperDARN lack adequate coverage in the dawn sector for this interval. Source data are provided as a Source Data file.

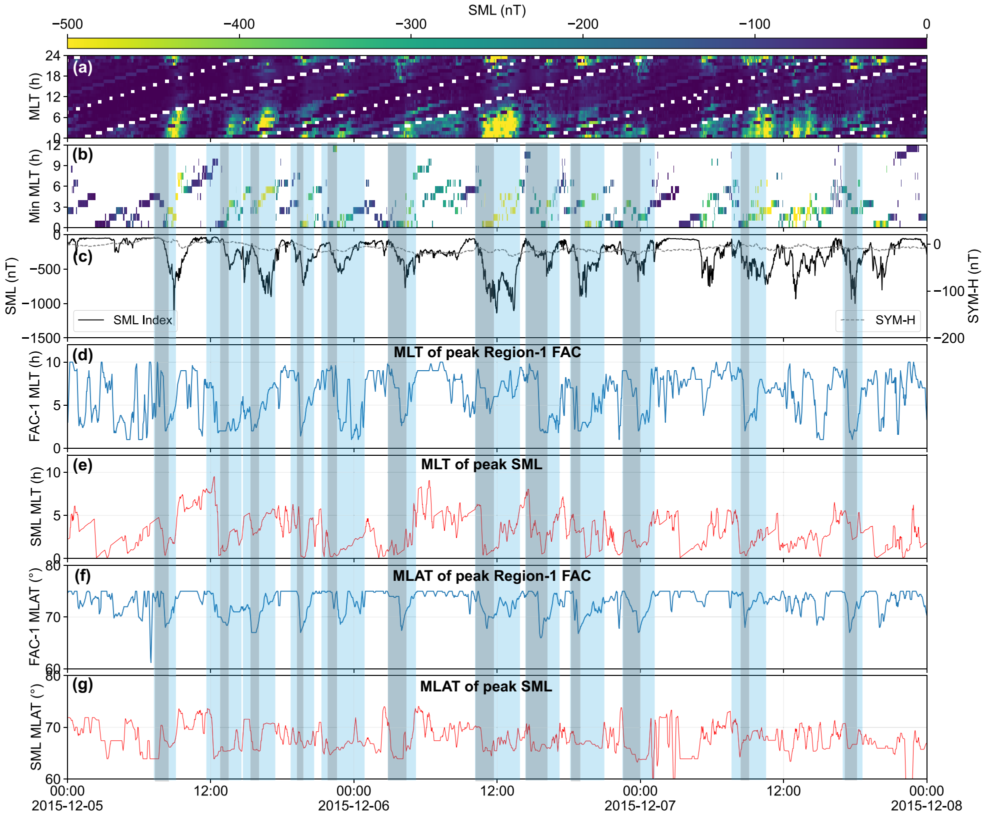

Supplementary Fig. 6 Substorm expansions and current peaks from Dec 05, 2015 to Dec 08, 2015. From top to bottom: **a** MLT-time distribution of regional SML and **b** Peak SML, **c** SML index and and SYM-H index, **d** MLT location of the peak upward Region 1 FAC and **e** peak westward AEJ on the dawnside, **f** MLAT location of the peak upward Region 1 FAC and **g** peak westward AEJ on the dawnside. Grey box correspond to substorm expansion phase. Blue boxes correspond to MLT-Mlat cycle of current peaks. Source data are provided as a Source Data file.

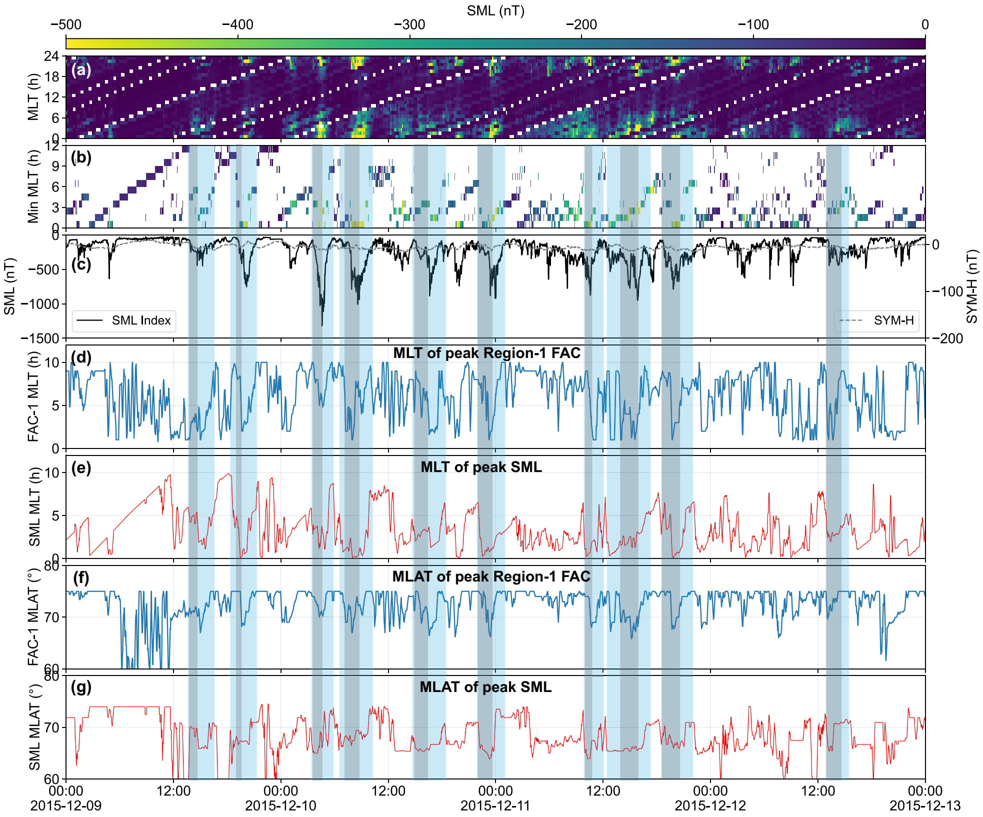

Supplementary Fig. 7 Substorm expansions and current peaks from Dec 9, 2015 to Dec 13, 2015. From top to bottom: **a** MLT-time distribution of regional SML and **b** Peak SML, **c** SML index and and SYM-H index, **d** MLT location of the peak upward Region 1 FAC and **e** peak westward AEJ on the dawnside, **f** MLAT location of the peak upward Region 1 FAC and **g** peak westward AEJ on the dawnside. Grey box correspond to substorm expansion phase. Blue boxes correspond to MLT-Mlat cycle of current peaks. Source data are provided as a Source Data file.

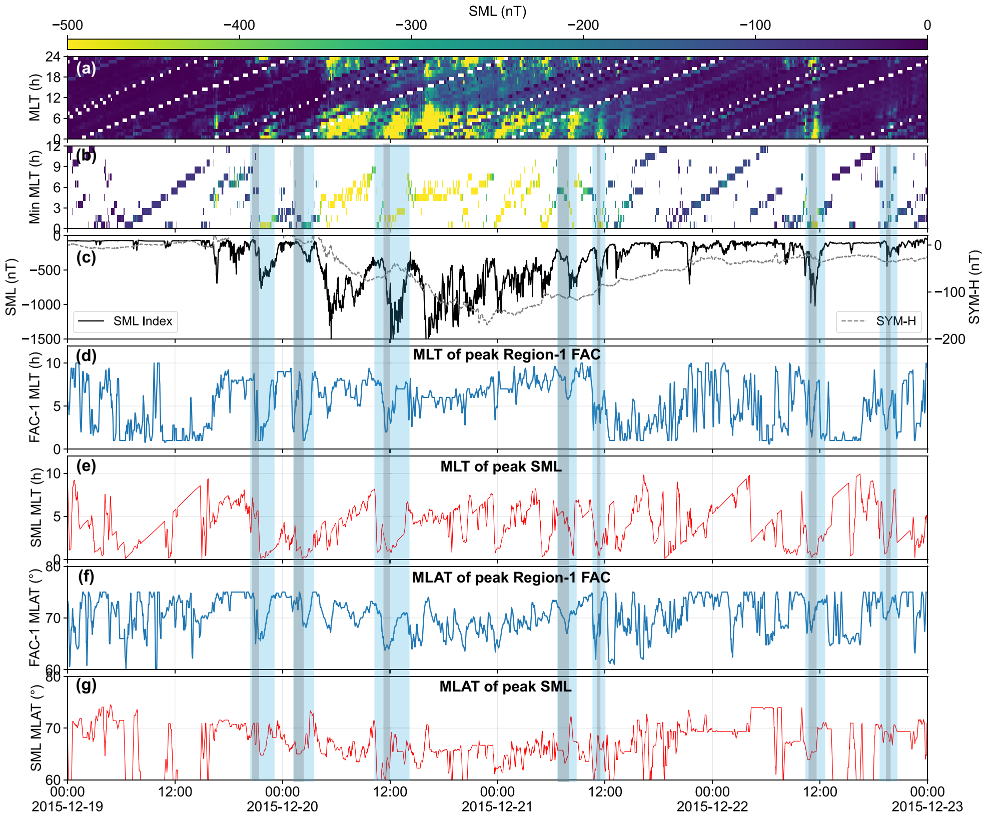

Supplementary Fig. 8 Substorm expansions and current peaks from Dec 19, 2015 to Dec 23, 2015. From top to bottom: **a** MLT-time distribution of regional SML and **b** Peak SML, **c** SML index and and SYM-H index, **d** MLT location of the peak upward Region 1 FAC and **e** peak westward AEJ on the dawnside, **f** MLAT location of the peak upward Region 1 FAC and **g** peak westward AEJ on the dawnside. Grey box correspond to substorm expansion phase. Blue boxes correspond to MLT-Mlat cycle of current peaks. Source data are provided as a Source Data file.
